# Manipulation of the Porcine Epidemic Diarrhea Virus Genome Using Targeted RNA Recombination

**DOI:** 10.1371/journal.pone.0069997

**Published:** 2013-08-02

**Authors:** Chunhua Li, Zhen Li, Yong Zou, Oliver Wicht, Frank J. M. van Kuppeveld, Peter J. M. Rottier, Berend Jan Bosch

**Affiliations:** 1 Institute of Animal Science and Veterinary Medicine, Shanghai Academy of Agricultural Sciences, Shanghai, P.R. China; 2 Virology Division, Department of Infectious Diseases and Immunology, Faculty of Veterinary Medicine, Utrecht University, Utrecht, The Netherlands; University of Kansas Medical Center, United States of America

## Abstract

Porcine epidemic diarrhea virus (PEDV) causes severe economic losses in the swine industry in China and other Asian countries. Infection usually leads to an acute, often lethal diarrhea in piglets. Despite the impact of the disease, no system is yet available to manipulate the viral genome which has severely hampered research on this virus until today. We have established a reverse genetics system for PEDV based on targeted RNA recombination that allows the modification of the 3′-end of the viral genome, which encodes the structural proteins and the ORF3 protein. Using this system, we deleted the ORF3 gene entirely from the viral genome and showed that the ORF3 protein is not essential for replication of the virus *in vitro*. In addition, we inserted heterologous genes (*i.e.* the GFP and *Renilla* luciferase genes) at two positions in the viral genome, either as an extra expression cassette or as a replacement for the ORF3 gene. We demonstrated the expression of both GFP and *Renilla* luciferase as well as the application of these viruses by establishing a convenient and rapid virus neutralization assay. The new PEDV reverse genetics system will enable functional studies of the structural proteins and the accessory ORF3 protein and will allow the rational design and development of next generation PEDV vaccines.

## Introduction

Porcine epidemic diarrhea virus (PEDV) causes diarrhea and dehydration in newborn piglets. The virus infects the epithelial cells of the small intestine resulting in severe mucosal atrophy and consequent malabsorption. PEDV is common and the cause of serious problems, particularly in pigs in Asia. The disease usually appears in winter during which it can cause high fatalities in suckling piglets (see for a recent review [1]). From 2010, an outbreak of PEDV has swept China with over 1 million fatalities among newborn piglets causing substantial economic losses in the swine industry [2]. The characteristics of the infection and its epidemiology were quite dramatic with morbidity and fatality approaching 100% in one-week old piglets, despite the use of commercial, inactivated vaccines. Virus transmission occurs via the fecal-oral route and possibly also by vertical transmission through lactation [2]. Currently there is no efficient way of treatment of the disease. Prevention of the infection usually relies on vaccination with cell culture adapted live-attenuated or inactivated viruses although the efficacy of current vaccines has been questioned [2], [3].

**Figure 1:**
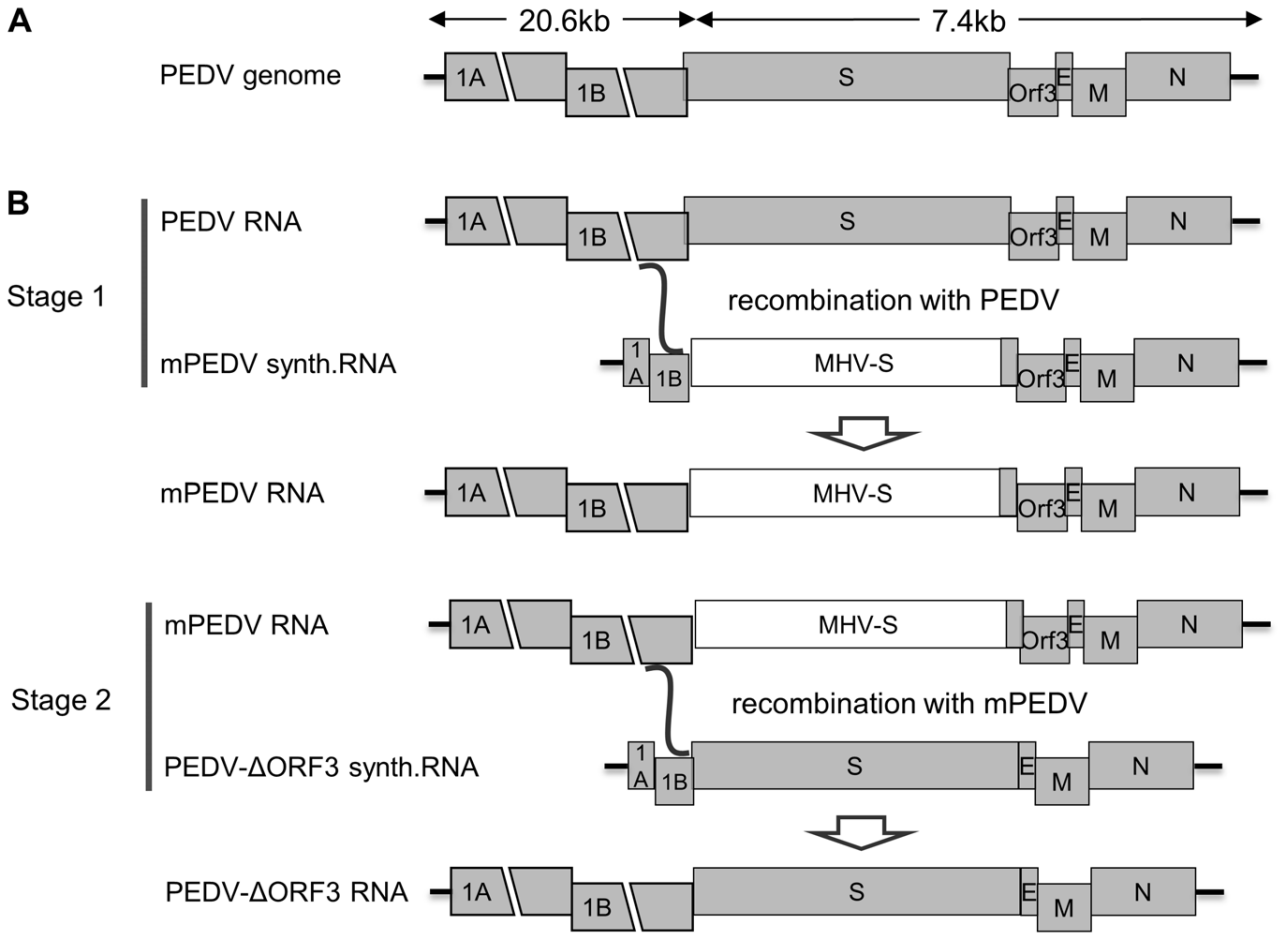
Coronavirus genome organization and targeted RNA recombination scheme. (A) Genomic organization of PEDV. (B) Targeted RNA recombination scheme to make the interspecies chimeric virus mPEDV (Stage 1) or recombinant PEDV derivatives e.g. lacking the ORF3 gene as shown here (Stage 2). The ectodomain-encoding region of the MHV S gene is shown as a light-grey box in the mPEDV genome. Synthetic RNAs transcribed from the transfer vectors (Fig. 2A) were electroporated into PEDV (Stage 1) or mPEDV (Stage 2) infected cells, respectively. A single recombination event (indicated by a curved line) anywhere within the 3′ region of ORF1b present in the donor RNA and viral genome generates a recombinant genome. Selection of recombinant progeny viruses against parental viruses was done on the basis of the acquired ability to form plaques in murine cell monolayers (Stage 1) or on the basis of the ability to infect VERO cells and the concomitantly lost ability to infect murine cells (Stage 2).

PEDV belongs to the alphacoronavirus genus within the *Coronavirinae* subfamily of the *Coronaviridae* family. Coronaviruses are important pathogens of concern for human and animal health. They occur in almost any species, usually causing respiratory or intestinal infections. Interest in these viruses has increased significantly as a result of the SARS epidemic in 2002 and 2003. Coronaviruses are enveloped viruses and possess a positive-sense RNA genome ranging from 26 to 32 kilobases, which is the largest viral RNA genome known (Fig. 1A). The 5′ two-third of the viral genome contains two large open reading frames (ORFs), 1a and 1b, which encode two non-structural polyproteins, pp1a and pp1ab, that direct genome replication and transcription. The remaining part of the genome contains ORFs specifying structural and non-structural proteins. They are expressed via a 3′-terminal nested set of subgenomic messenger RNAs, the transcription of which is regulated by conserved six-nucleotides transcription-regulating sequences (TRSs; in PEDV XUA(A/G)AC [4]). These subgenomic mRNAs encode at least four structural proteins, three membrane anchored proteins called the spike (S), membrane (M) and envelope (E) protein, and the nucleocapsid (N) protein that encapsidates the genomic RNA. The non-structural proteins expressed from the subgenomic mRNAs encode one or more accessory proteins, which are specific for each coronavirus genus. The genome structures of alphacoronaviruses including PEDV and related members such as the human coronavirus (hCoV) strains 229E and NL63 show the typical set of essential core genes but they share only one accessory gene, ORF3, located between the S and the E gene (Fig. 1A). The PEDV ORF3 gene encodes a 224 amino acids (aa) long protein with three to four predicted transmembrane domains [5].

Entry of coronaviruses into their host cells is mediated by the approximately 200 kDa large S glycoprotein. Trimers of S form the characteristic spikes on the viral surface which interact with the host receptor and mediate membrane fusion. PEDV was reported to utilize the porcine aminopeptidase N as a receptor [6]. Yet, PEDV is usually propagated in VERO cells, which are derived from the African green monkey kidney, indicating that PEDV can utilize non-porcine receptors for cell entry. Propagation of PEDV in cell culture requires addition of trypsin which is believed to prime or activate the S protein for membrane fusion during virus cell entry and syncytia formation [7] Recently it was demonstrated that trypsin cleavage may also play a role in detachment of the virus from infected cells [8]. Interestingly, a cell culture adapted strain was reported to replicate in the absence of trypsin [9], which suggests that the virus acquired mutations in the S protein conferring its trypsin-independence. The S protein also stimulates the induction of neutralizing antibodies and hence is an important target in developing effective vaccines.

Research on the molecular biology and pathogenicity of PEDV has been severely hampered by the lack of a reverse-genetic system. Here we report the first reverse genetic system for PEDV based on targeted RNA recombination. Establishment of the reverse genetic system included two stages (Fig. 1B). One was the generation of the chimeric virus mPEDV, a PEDV derivative carrying spikes derived from the murine coronavirus mouse hepatitis virus (MHV), hence growing only in murine cells. In the second stage the mPEDV virus was used as a recipient virus to reintroduce the PEDV spike along with other genome alterations, *in casu* the deletion of the ORF3 gene or the insertion of foreign, reporter genes. The generated PEDV derivatives now carrying again PEDV spikes could be easily selected by their regained tropism for non-murine cells.

## Results

**Figure 2:**
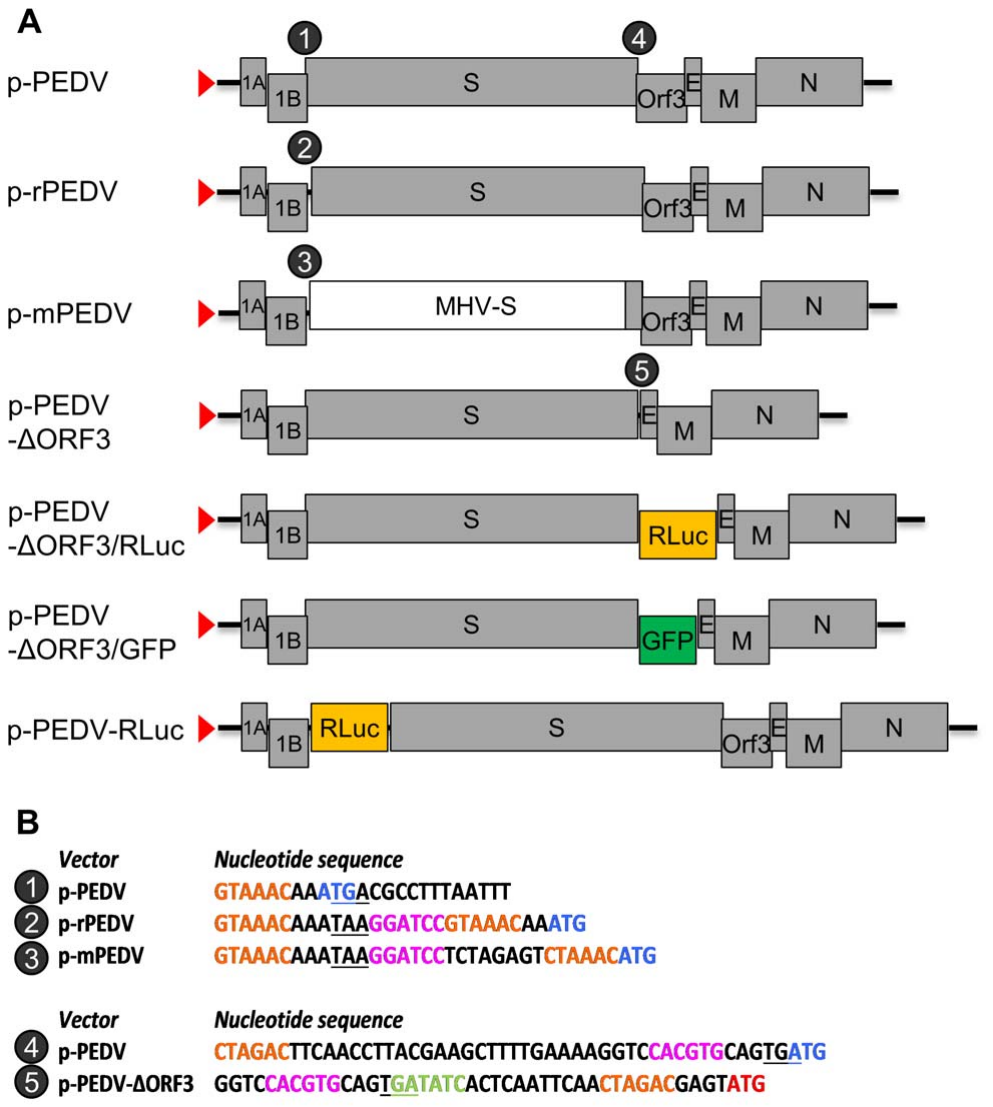
PEDV transfer vectors. (A) The pPEDV transfer vector contains the 5′-proximal 605 nt fused to the 3′ approximately 8 kilobases of the PEDV genome. All other vectors are derivatives thereof. The red triangle indicates the T7 promoter in the transfer vectors from which synthetic RNAs were made *in vitro* using T7 RNA polymerase. (B) Nucleotide sequences of junctions in the PEDV transfer vectors. Encircled numbers correspond to the numbered positions in the vector maps as indicated in Fig. 2A. (upper panel) The stop codon of ORF1b is underlined, the start codon of S is in blue, the transcription regulatory sequences (XUA(A/G)AC; [4]) are in orange and the *Bam*HI site is indicated in purple. (lower panel) The stop codon of the S gene is underlined, the start codon of the ORF3 gene is in blue, the start codon of E gene is in red, the transcription regulatory sequences are in orange and the unique *Pml*I and *Eco*RV sites are indicated in purple and green, respectively.

To set up a targeted RNA recombination system for PEDV we first created a recombinant PEDV virus carrying MHV spikes (mPEDV). To this end a transfer vector p-mPEDV was construced (Fig. 2A) that was composed of a 5′-terminal genomic cDNA fragment ligated to a cDNA representing the entire 3′-terminal part of the genome starting within ORF1b, except for the S gene. This gene was replaced by a hybrid gene encoding a chimeric S protein composed of the 1,263 aa long ectodomain from MHV S and the transmembrane domain plus cytoplasmic tail (61 aa) from PEDV S. RNA was transcribed from the T7 promotor of this vector and electroporated into PEDV-infected VERO cells after which the cells were overlaid onto a murine cell (L cells) monolayer. The recombinant mPEDV virus generated during subsequent incubation was cloned by two rounds of plaque selection on L cells.

**Figure 3:**
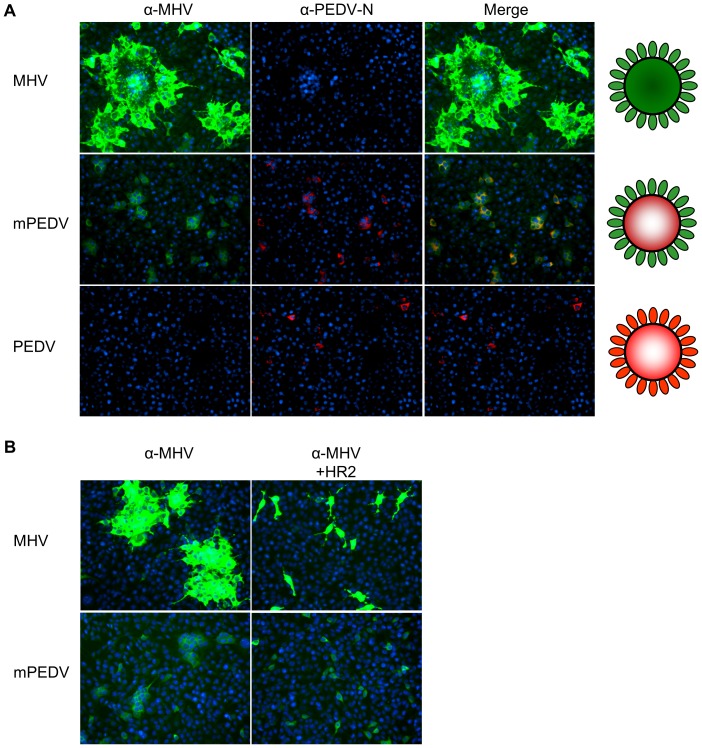
Characterization of a chimeric PEDV carrying MHV spikes. (A) Immunofluorescence analysis of mPEDV, MHV and PEDV infected cells. L cells infected with mPEDV were fixed and double immunolabeled with a polyclonal antibody against MHV (green) and a monoclonal antibody against the PEDV nucleocapsid (red). MHV and PEDV infected L cells were taken along for comparison. Nuclei are visualized with DAPI (blue). Overlay pictures (Merge) and graphical presentation of the MHV, mPEDV and PEDV virions are indicated at the right. Of note, the α-MHV fluorescence signal for MHV is significantly stronger than that for mPEDV due to the contribution of antibodies directed against other MHV proteins in the polyclonal MHV serum. (B) Inhibition of syncytia formation by the MHV-S HR2-peptide fusion inhibitor. HR2 peptide (4 µM; [32]) was added to MHV and mPEDV infected cells at 2 hours p.i. and kept present until 6.5 hours p.i. when cells were fixed and immunolabeled with the polyclonal MHV serum (green). Nuclei are visualized with DAPI (blue).

The identity of purified mPEDV viruses was checked at a genetic level by RT-PCR sequencing of the ORF1b-S gene junction (data not shown) and at the protein level by an immunofluoresence assay (Fig. 3A). All mPEDV infected cells stained positive both with the polyclonal MHV serum and with the monoclonal antibody directed against the PEDV nucleocapsid protein confirming the purity and the identity of the chimeric virus. In contrast to the parental virus, mPEDV displayed the ability to induce syncytia in the absence of trypsin (Fig. 3A). As predicted, cell-cell fusion mediated by mPEDV could be inhibited by a MHV S specific, peptidic fusion inhibitor (Fig. 3B).

**Figure 4:**
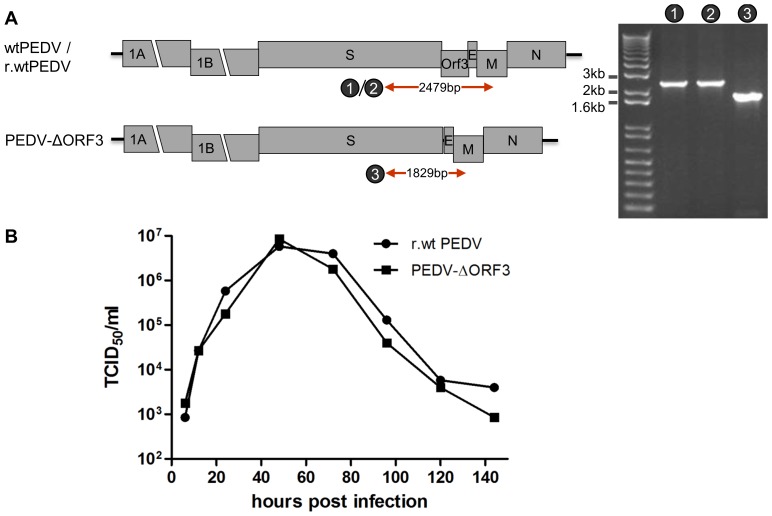
Characterization of a PEDV recombinant virus lacking ORF3. (A) Genetic analysis of PEDV-ΔORF3. RT-PCR was performed covering the S-ORF3-E-M region (primers 4538/4977) using RNA templates isolated from wtPEDV, r-wtPEDV and PEDV-ΔORF3, and analyzed by gel electrophoresis. The expected sizes of the RT-PCR products (numbered 1 to 3) are indicated in the genome maps. For primer sequences, see Table 1. (B) Multi-step growth kinetics of r-wtPEDV and PEDV-ΔORF3. VERO cells were infected with each recombinant PEDV (MOI = 0.01), washed after three hours and viral infectivity in the culture media was determined at different times p.i. by a quantal assay on VERO cells from which TCID_50_ values were calculated.

The generated mPEDV virus was used as a recipient virus to reintroduce by similar procedures the PEDV spike along with other genome modifications by targeted RNA recombination. Candidate recombinant viruses carrying the PEDV spikes can be selected by their regained ability to replicate in VERO cells. Apart from the wild-type recombinant virus (r-wtPEDV) we aimed at constructing a virus lacking the ORF3 gene (PEDV-ΔORF3). A number of cell culture adapted viruses including the strain used in this study have each acquired during passaging an identical 51 nucleotide in-frame deletion in the ORF3 gene, giving rise to a 17 amino acid deletion (aa 82–98) in their ORF3 protein [10]. We constructed a transfer vector (pPEDV-ΔORF3, Fig. 2A) from which the entire ORF3 gene was deleted. Donor RNAs transcribed from the pPEDV and pPEDV-ΔORF3 transfer vectors were electroporated into mPEDV-infected L cells after which we were able to recover and purify the r-wtPEDV and PEDV-ΔORF3 viruses in VERO cells. RT-PCR analysis confirmed the intended loss of the ORF3 gene from the viral genome (Fig. 4A) and the genetic identity of the ORF3 lacking virus was further verified by sequencing of the RT-PCR product (data not shown). The PEDV-ΔORF3 grew unimpaired in cell culture (Fig. 4B), demonstrating that the ORF3 gene product is not required for virus propagation *in vitro*. In addition, the successful deletion of the ORF3 gene from the viral genome demonstrated the feasibility of the mPEDV-based targeted RNA recombination system to manipulate the 3′ end of the viral genome.

**Figure 5:**
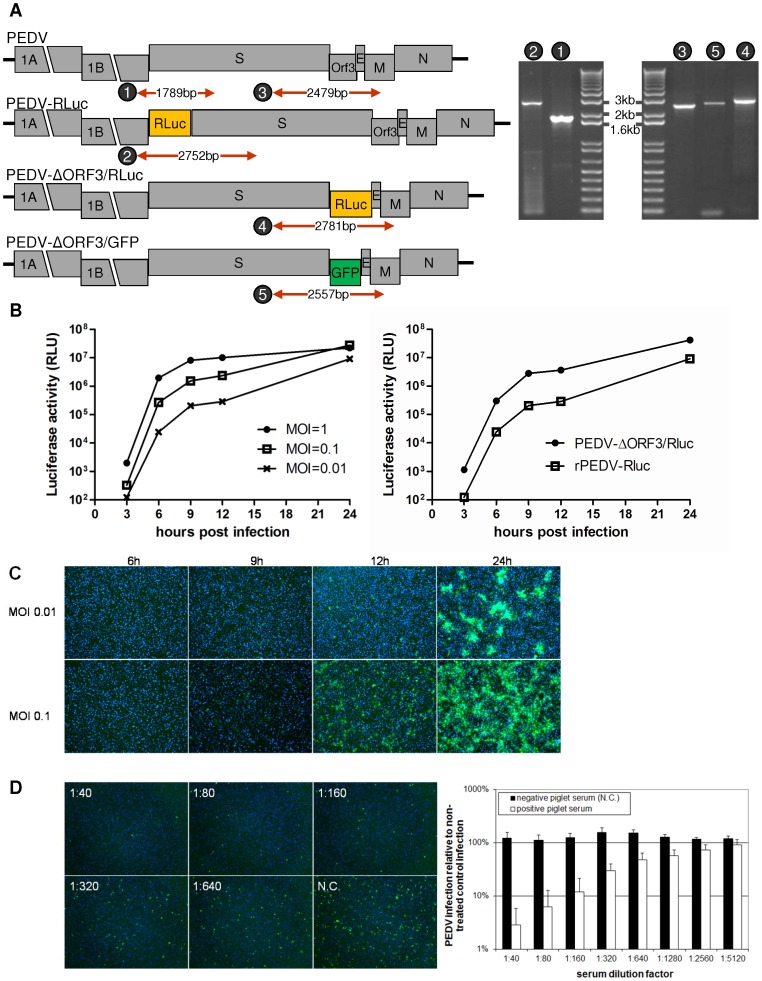
Recombinant PEDVs carrying *Renilla* luciferase and GFP genes. (A) Genetic analysis of recombinant viruses. RT-PCR was performed covering the 1b-S junction (primers 5109/4535) or the S-M region (primers 4538/4977) using RNA templates isolated from wild-type PEDV, PEDV-Rluc, PEDV-ΔORF3/Rluc or PEDV-ΔORF3/GFP, and analyzed by gel electrophoresis. The expected sizes of the RT-PCR products (numbered 1 to 5) are indicated in the genome maps. For primer sequences, see Table 1. (B) Luciferase expression by the recombinant PEDV-Rluc and PEDV-ΔORF3/Rluc viruses. Left panel: VERO cells were infected with PEDV-Rluc at an MOI of 0.01, 0.1 or 1. Right panel: VERO cells were infected with PEDV-Rluc and PEDV-ΔORF3/Rluc at an MOI of 0.01. Intracellular *Renilla* luciferase activity (y-axis; Relative Light Units [RLU]) was determined at different times postinfection. (C) GFP expression by the recombinant PEDV-ΔORF3/GFP virus. VERO cells were infected with PEDV-ΔORF3/GFP at an MOI of 0.01 or 0.1 and fluorescence images were taken at different times p.i. Nuclei of cells were stained with DAPI (blue). (D) A rapid virus neutralization assay based on recombinant PEDVs expressing reporter proteins. PEDV-ΔORF3/Rluc and PEDV-ΔORF3/GFP (8,000 TCID50) were mixed with subsequent dilutions of serum positive for PEDV antibodies and a negative control serum (N.C.) for 30 minutes at room temperature. Mixtures were incubated with VERO cells and *Renilla* luciferase (left panel) or GFP (right panel) expression was measured at 8 and 9 hours p.i., respectively.

We next explored the possibilities of expressing heterologous proteins from the PEDV genome by inserting reporter genes at different genomic positions. Transfer vectors were made with the *Renilla* luciferase gene (936 nt) and the GFP gene (720 nt) at the position of ORF3, creating the pPEDV-ΔORF3/Rluc and pPEDV-ΔORF3/GFP vectors (Fig. 2A). These marker genes are under the transcriptional control of the TRS of ORF3 (CTAGAC) which is located in the 3′end of the S gene, 46 nucleotides upstream of the ORF3 gene. The *Renilla* luciferase gene was also inserted as an extra expression cassette between the ORF1b and S gene, creating the pPEDV-Rluc vector. To this end the otherwise overlapping ORFs 1b and S were first separated and a unique *Bam*HI restriction site was introduced (p-rPEDV vector, Fig. 2A and B), which did not hamper the generation of a viable virus (data not shown). The *Renilla* luciferase gene was subsequently cloned into the *Bam*HI site of the p-rPEDV vector under control of the TRS in ORF1B (GTAAAC) originally driving S gene expression, whereas the S gene was provided with a new TRS (GTAAAC; Fig. 2B). The PEDV-ΔORF3/GFP, PEDV-ΔORF3/Rluc and PEDV-Rluc recombinant viruses were successfully recovered by the targeted RNA recombination procedure. RT-PCR analyses confirmed the insertion of both reporter genes at the intended positions (Fig. 5A), which was further confirmed by sequencing.

We studied the luciferase expression by the 2 recombinant viruses carrying a Rluc gene as well as the expression kinetics of one of these viruses, PEDV-Rluc, upon infection of VERO cells at three different MOI’s. The result shows (Fig. 5B) that luciferase expression levels were linearly related to the MOI during the early phase of infection until 12 hours p.i. whereas at 24 hours p.i. luciferase values converged due to reinfections. Similar kinetics of luciferase expression, but to higher levels, was observed for the PEDV-ΔORF3/Rluc recombinant virus (Fig. 5B). Next we studied the GFP expression of the PEDV-ΔORF3/GFP virus upon infection of VERO cells at two MOI’s. GFP expression in PEDV-ΔORF3/GFP virus infected cells could be seen starting from 9 hours p.i. and became clearly evident at 12 hours p.i. (Fig. 5C). The cell adapted PEDV DR13 p100 strain can propagate in the absence of trypsin in the growth medium but does not form syncytia when trypsin is absent. Yet the clustered appearance of GFP-positive cells suggests that the virus predominantly spreads locally from cell to cell which may correlate with the reported cell surface attachment of progeny viruses released from infected cells in the absence of trypsin [11].

The early detection of the luciferase and GFP reporter proteins during infection can be applied to develop a more rapid PEDV neutralization diagnostic test. The readout of the classical virus neutralization assay with wild-type PEDV is based on the visual inspection of cytopathic effect and can only be done after a multicycle infection which takes at least 2–3 days. Thus, the PEDV-ΔORF3/GFP and PEDV-ΔORF3/RLuc virus were preincubated with dilutions of serum obtained from an experimentally PEDV-infected pig and control serum, and the mixtures were subsequently added to VERO cells and incubated after which the GFP and *Renilla* luciferase expression was recorded at 9 and 6 hours p.i., respectively (Fig. 5D). In contrast to the control serum, the PEDV antibody-positive serum was able to neutralize PEDV infection as reflected by the reduction of GFP positive cells and luciferase activity. The results demonstrate that neutralization of the PEDV-ΔORF3/GFP and PEDV-ΔORF3/RLuc virus can already be scored within a single replication cycle, thereby significantly speeding up the assay time. This type of assay is additionally preferred as it avoids the subjectivity that is associated with scoring of cytopathic effects.

## Discussion

Here we describe the first reverse genetics system for PEDV. As we illustrate, this system now enables the manipulation of the 3′proximal ∼8 kilobases of the PEDV genome including the structural protein genes. Generation of PEDV recombinants was based on the well-known high efficiency of RNA recombination of coronaviruses in combination with host cell tropism switching for selection of the recombinant viruses. Similar recombination systems have been successfully developed for MHV and FIPV coronaviruses by the Masters and Rottier laboratories [12], [13]. For a number of coronaviruses genetic engineering of the full length genome has also become accomplished by the development of infectious cDNA clones [14]–[20]. The ability to manipulate the PEDV genome will be extremely valuable to study the molecular and biological features of PEDV infections as well as to develop new tools and strategies for prevention and therapy of this important veterinary pathogen.

Unlike most other coronaviruses, the PEDV genome contains only a single accessory gene, the ORF3 gene, which encodes a multispanning 224-aa long membrane protein. Intriguingly, propagation of PEDV isolates in tissue culture cells readily leads to deletions within ORF3 suggesting a dispensable role, at least for the parts deleted from the ORF3 protein, for viral replication *in vitro*. In all these adapted viruses a shorter ORF3 gene product is still translated with a minimal size of 91 amino acids [10]. The ORF3 gene of the cell-adapted DR13 vaccine strain (GenBank accession no.: JQ023162.1) employed in our study has a 49 nucleotide deletion compared to that of the parental DR13 virus (GenBank accession no.: JQ023161.1), but still encodes the N-terminal 81 residues long ORF3 protein part including the first transmembrane domain, after which it gets out of frame due to the deletion. The deletion of the entire ORF3 gene from the genome did not have any obvious effect on viral propagation *in vitro*, demonstrating that this ∼10 kD polypeptide does not serve an essential function during replication in culture cells.

The function of the PEDV ORF3 product remains enigmatic. Recently it was shown that the protein exhibits ion channel activity and modulates virus production [5]. siRNA knockdown of ORF3 gene in PEDV infected cells reduced the number of particles released from the cells [5]. The question remains here why passaging of PEDV in cell culture would lead to the functional loss of a gene beneficial for virus propagation *in vitro*, unless the 91-residue truncated protein still provides that function. Homologues of the ORF3 protein are found in all other alphacoronaviruses. The ORF3 protein of hCoV-NL63 was shown to be N-glycosylated at the amino terminus and incorporated into virions [21]. Yet, deletion of the ORF3 gene from the viral genome had little effect on virus replication in cell culture [22]. Like for PEDV, loss of ORF3 genes of the alphacoronaviruses TGEV and hCoV-229E (here named ORF4) is associated with unimpaired virus passaging in cell culture [23], [24]. Despite a non-essential role in cell culture, the maintenance of the ORF3 gene in alphacoronavirus field isolates strongly points to an important role of the ORF3 protein in natural infection in the animal host. Consistently, the loss of virulence of life-attenuated PEDV vaccine strains has been associated with mutations in the ORF3 gene resulting from cell culture adaptation [10], [25] although a contribution of the numerous additionally acquired mutations in other genes such as the spike gene can obviously not be excluded [26], [27]. The specific function of the ORF3 protein (and other viral proteins in the 3′ genome region) in PEDV replication and pathogenesis can now be further investigated using the reverse genetics system.

The introduction of foreign genes at different genomic positions without apparent great fitness loss of the virus *in vitro* (data not shown) once more illustrates the remarkable genome plasticity of the coronavirus genome [28], [29]. The insertion of reporter genes like for GFP and luciferase will be very useful for the study of various molecular and virological aspects of PEDV infection. In addition, as we demonstrate here, these reporter properties may also be exploited for applications such as the establishment of convenient virus neutralization assays that provide answers within hours rather than days. Furthermore, genomic insertion of genes encoding foreign antigens using the reverse genetics system opens avenues to the development of PEDV as a vaccine vector for protection against other relevant porcine pathogens in addition to PEDV.

## Materials and Methods

### Cells, Viruses and Antibodies

L [12] and VERO CCL81 cells (purchased from ATCC) were maintained as monolayer cultures in Dulbecco’s modified Eagle medium containing 10% fetal calf serum, 100 IU of penicillin/ml, and 100 µg of streptomycin/ml (all from Life Technologies, Ltd., Paisley, United Kingdom). PEDV (isolated from a commercial vaccine of GreenCross, South Korea) was propagated in Vero cells in the absence of trypsin. Virus was harvested by three cycles of freeze-thawing the infected cells and supernatant followed by removal of cell debris by centrifugation at 3,000×*g* for 20 minutes. Virus infectivity in the supernatant was measured by an end-point dilution assay on VERO cells and 50% tissue culture infectious doses (TCID_50_) were calculated. MHV (strain A59) was propagated in mouse L cells as described previously [12]. The rabbit anti-MHV serum K135 raised against purified MHV has been described elsewhere [30]. The monoclonal antibody (MAb) 3F12 recognizing the PEDV nucleocapsid protein was obtained from BioNote, Korea. Polyclonal PEDV serum from a pig experimentally infected with PEDV (strain CV777) was kindly provided by Dr. Kristin van Reeth (Gent University). PEDV antibody-negative control serum was obtained from a newborn piglet deprived of colostrum.

### Construction of pPEDV Transfer Vector and Derivatives

#### pPEDV vector

A cDNA clone encompassing the 3′-terminal 7,832 nt part of the PEDV genome starting within ORF1b was obtained by reverse transcription-PCR (RT-PCR) with viral genomic RNA isolated from virions as a template and primers 4922 and 4815 as plus- and minus-strand primers (for primer sequences see Table 1), respectively. The overhang of primer 4922 and primer 4815 contained a *Bgl*II and a *Pac*I restriction site, respectively. The *Bgl*II-*Pac*I digested fragment was cloned into the *Bam*HI-*Pac*I digested pMH54 vector [12], creating the plasmid pPEDV-1b-3T. The 5′-terminal 605 nt of ORF1a was amplified using primers 4884 and 4885. Primer 4884 contains a T7 polymerase recognition site, as well as a *Bgl*II restriction site and primer 4885 contained a *Bam*HI restriction site. The *Bgl*II-*Bam*HI digested fragment was ligated into the *Bam*HI site of the pPEDV-1b-3T plasmid, resulting in the pPEDV vector.

**Table 1:** Primers.

Primer	Location^a^ (nucleotides)	Sense	Sequence (5′-3′)^b^
4535	S/22164–22187	−	GCCGCAGAGACAGTAATATTAACA
4538	S/23484–23507	+	GTATAGTGCGTCTCTCATCGGTGG
4814	S/24603–24624	+	GTGG**CCTTGG**TGGGTTTGGTTG
4815	3′UTR/28012–28033	−	GC**TTAATTAA**TTTTTTTTTGTGTATCCATATCAACACCGTC
4884	5′UTR/1–25	+	GC**AGATCT**TAATACGACTCACTATAGGGACTTAAAAAGATTTTCTATCTACGG
4885	1A/584–605	−	**GGATCC** GAGCTCTAACTCTTCGAGGAAG
4886	1B/20156–20176	+	**GGATCC** GAGAACGTGTCTAAAGAAGGC
4921	T7/N.A.	+	GC**AGATCT**TAATACGACTCACTATAGGG
4922	1B/20156–20173	+	GC**AGATCT**GAGAACGTGTCTAAAGAAGG
4923	1B/20618–20649	−	GC**GGATCC**TTATTTGTTTACGTTGACCAAATG
4924	E/25655–25674	−	GC**CGGCCG**AGATCTTTATATGTCAATAACAGTAC
4977	M/25942–25962	−	ATTATCCACAGCATAAGAGTG
5109	1B/20396–20416	+	GACGGCAACACCATGCATGCC
5127	S/20629–20655	+	**GGATCC** GTAAACAAATGACGCCTTTAATTTAC
5300	E/25403–25446	+	GGTC**CACGTG**CAGT**GATATC**ACTCAATTCAACTAGACGAGTATG
5301	N/26458–26477	−	GCGAGTA**CCTTAGAAAGG**GG

a The location of primers is relative to the full genome sequence of the PEDV CV777 strain (GenBank accession No. AF353511).

b Endonuclease restriction sites used for cloning are indicated in bold.References.

#### p-rPEDV vector

A transfer vector was constructed in which the partly overlapping ORF1b and S gene were separated by introduction of a unique *Bam*HI site to facilitate further cloning. The stop codon of ORF1b was mutated to TAA to knock out the overlapping ATG start codon of the spike gene. First, the forward primer 5127 containing the *Bam*HI site and a TRS (TAAAC), and the reverse primer 4815 containing a unique *Pac*I site were used to amplify the 3′ proximal 7,332 nt of the PEDV genome starting with the spike gene. This fragment was cloned into the *Bam*HI-*Pac*I site of pMH54 vector, creating the pPEDV-S-3T vector. Second, primers 4884 and 4885 containing a *Bgl*II and *Bam*HI site, respectively, were used to RT-PCR amplify the ORF1a fragment which was introduced into the *Bam*HI digested pPEDV-S-3T vector creating the pPEDV-1a-S-3T plasmid. Third, primers 4922 and 4923 that contain a *Bgl*II and *Bam*HI in the overhang, respectively, were used to amplify the ORF1b fragment by RT-PCR. This fragment was cloned into the *Bam*HI site of the pPEDV-1a-S-3T vector, creating the p-rPEDV vector.

#### p-mPEDV vector

First, the plasmid pTUMS [31] encoding the MHV spike was used as an intermediate vector to construct a chimeric spike composed of the ectodomain of MHV and the transmembrane and cytoplasmic domain of PEDV. For the construction of the hybrid gene, a *Sty*I restriction site was used that is located in both S genes at the transition between the protein’s ectodomain and transmembrane domain. The forward primer 4814 (*Sty*I site in overhang) and reverse primer 4924 (*Eag*I site in overhang) were used to amplify the 3′ end of the PEDV S gene and downstream sequences and cloned into the *Sty*I-*Eag*I digested pTUMS plasmid, creating the pTUMS(MP) vector. Second, to create the p-mPEDV vector, the PEDV S gene in the p-rPEDV vector was replaced by the chimeric MHV-PEDV spike gene by cloning the *Bam*HI-*Pml*I digested fragment of pTUMS(MP) into the *Bam*HI-*Pml*I digested p-rPEDV vector.

#### pPEDV-ΔORF3 vector

Primers 5300 and 5301 were used to amplify the E gene and downstream sequences using the pPEDV vector as a template. The forward primer 5300 contained a *Pml*I and an *Eco*RV restriction site and the reverse primer 5301 contained an restriction *Eco*NI site to facilitate further cloning. The *Pml*I-*Eco*NI digested PCR fragment was cloned into the *Pml*I-*Eco*NI digested pPEDV vector to create the pPEDV-ΔORF3 vector.

#### pPEDV-RLuc and pPEDV-ΔORF3/RLuc vector

The *Renilla* luciferase gene was excised from the pRLnull vector (Promega) using enzymes *Nhe*I and *Xba*I, blunted with DNA-polymerase I large (Klenow) fragment and ligated into the *Bam*HI digested and blunted p-rPEDV vector or the *Eco*RV digested pPEDV-ΔORF3 vector, resulting in the pPEDV-RLuc and pPEDV-ΔORF3/RLuc transfer vector, respectively.

#### pPEDV-ΔORF3/GFP vector

The GFP gene was excised from the pEGFP-N1 plasmid (Clontech) with enzymes *Nco*I and *Not*I, blunted with DNA-polymerase I large (Klenow) fragment and ligated into the *Eco*RV digested pPEDV-ΔORF3 vector yielding the pPEDV-ΔORF3/GFP transfer vector.

The identity of all generated transfer vectors was verified by sequencing.

### Targeted RNA Recombination

A targeted recombination system was established for PEDV in a two-stage process as outlined in Fig. 1B.

#### Stage 1 Generation of mPEDV

Introduction of the hybrid MHV-PEDV S gene into the PEDV genome by targeted RNA recombination was carried out essentially as described previously for MHV and FIPV [12], [13]. Briefly, capped runoff donor RNA transcripts were synthesized from the *Pac*I-linearized p-mPEDV vector using a T7 RNA polymerase kit (Ambion) as specified by the manufacturer. Donor RNA was electroporated (Gene Pulser electroporation apparatus [Bio-Rad]; two consecutive pulses of 0.3 kV/975 µF) into PEDV-infected (multiplicity of infection [MOI] of 0.4) VERO cells (2×10^7^ cells) at 8 hours post infection (p.i.). The electroporated cells were co-cultured in a 25-cm^2^ flask with 5×10^6^ murine L cells. After 48–60 h of incubation at 37°C, when syncytia could be detected in the murine L cells, progeny virus in the culture supernatant was harvested and mPEDV recombinant virus was purified by two consecutive cycles of plaque purification on L cells at 37°C.

#### Stage 2 Generation of recombinant PEDVs

The construction of PEDV recombinant viruses that had regained the PEDV S gene was carried out in a reverse process by using pPEDV-derived donor RNAs and mPEDV as the recipient virus. Capped runoff transcripts were synthesized from *Pac*I-linearized pPEDV, pPEDV-Rluc, pPEDV-ΔORF3, pPEDV-ΔORF3/Rluc, or pPEDV-ΔORF3/GFP, respectively, with a T7 RNA polymerase kit (Ambion) as specified by the manufacturer. The donor transcripts were electroporated (as specified above) into murine L cells (2×10^7^ cells) that had been infected 4 h earlier with mPEDV (MOI = 1). These cells were then plated onto a monolayer of VERO cells. After 4–5 days of incubation at 37°C progeny virus in the culture supernatant was harvested by freeze-thawing and candidate recombinant viruses were purified by two rounds of end-point dilutions on VERO cells. Recombinant genotypes were confirmed by RT-PCR on purified genomic RNA and subsequent sequencing.

### (Immuno)fluorescence Microscopy

L cells and VERO cells were inoculated with MHV, mPEDV or PEDV (MOI = 0.05). After 2 hours of incubation the cells were washed with PBS and incubated in culture medium. At 6.5 hours p.i., the cells were rinsed with PBS and fixed with 3.7% formaldehyde for 20 min at room temperature. The cells were washed three times with PBS and incubated with the K135 rabbit-α-MHV serum and the 3F12 mouse MAb α-PEDV-N. After 30 min at room temperature, the cells were rinsed three times with PBS and stained with goat α-rabbit FITC-conjugated and donkey-α-mouse Cy3 conjugated secondary antibodies (Cappel). Nuclei were stained with DAPI (Molecular Probes) for 10 min at room temperature. Finally, the cells were washed three times with PBS and fluorescence was viewed with an EVOS-fl fluorescence microscope (Advanced Microscopy Group) at 10× magnification. The EVOS-fl was also used to view GFP fluorescence from PEDV-ΔORF3/GFP infected cells after paraformaldehyde fixation.

### *Renilla* Luciferase Assay

VERO cell monolayers were infected as described above with the PEDV-Rluc and PEDV-ΔORF3/Rluc viruses at indicated MOI’s. At indicated times post infection, cell lysate samples were assayed for luciferase activity using the *Renilla* Luciferase Assay system (Promega) according to the manufacturer’s instructions, and the relative light units (RLU) were determined with a Berthold Centro LB 960 plate luminometer.

### Virus Neutralization Assay

PEDV-ΔORF3/Rluc or PEDV-ΔORF3/GFP were mixed with serial dilutions of positive or negative piglet serum or with cell culture medium. The inoculum was incubated for 30 minutes at room temperature to allow virus neutralization before inoculating VERO cell monolayers as described above. Cells were either lysed at 8 hours post infection and assayed for *Renilla* luciferase activity as described above or subjected to fluorescence microscopy as described above at 9 hours post infection.
